# Abnormal effective connectivity in visual cortices underlies stereopsis defects in amblyopia

**DOI:** 10.1016/j.nicl.2022.103005

**Published:** 2022-04-08

**Authors:** Xia Chen, Meng Liao, Ping Jiang, Huaiqiang Sun, Longqian Liu, Qiyong Gong

**Affiliations:** a Department of Optometry and Visual Science, West China Hospital, Sichuan University, Chengdu, China; b Department of Ophthalmology, West China Hospital, Sichuan University, Chengdu, China; c Huaxi MR Research Center (HMRRC), Department of Radiology, West China Hospital of Sichuan University, Chengdu, China; d Research Unit of Psychoradiology, Chinese Academy of Medical Sciences, Chengdu, China; e Functional and Molecular Imaging Key Laboratory of Sichuan Province, Chengdu, China; f Imaging Research Core Facilities, West China Hospital of Sichuan University, Chengdu, Sichuan, China

**Keywords:** Amblyopia, Stereopsis, Resting-state fMRI, Effective connectivity, Spectral dynamic causal modeling, Perceptual learning, MT, middle temporal area, MST, medial superior temporal area, LO, lateral occipital, IPS, intraparietal sulcus, fMRI, functional magnetic resonance imaging, ROIs, regions of interest, DCM, dynamic causal modeling, EC, effective connectivity, HCs, healthy controls, PEB, parametric empirical Bayes, SD, standard deviation, M, male, F, female, TNO, the Netherlands Organization for applied scientific research, BCVA, best-corrected visual acuity, TR, repetition time, TE, echo time, MNI, Montreal Neurological Institute, BOLD, blood oxygen level-dependent, Pp, posterior probability, LOC, lateral occipital complex

## Abstract

The neural basis underlying stereopsis defects in patients with amblyopia remains unclear, which hinders the development of clinical therapy. This study aimed to investigate visual network abnormalities in patients with amblyopia and their associations with stereopsis function. Spectral dynamic causal modeling methods were employed for resting-state functional magnetic resonance imaging data to investigate the effective connectivity (EC) among 14 predefined regions of interest in the dorsal and ventral visual pathways. We adopted two independent datasets, including a cross-sectional and a longitudinal dataset. In the cross-sectional dataset, we compared group differences in EC between 31 patients with amblyopia (mean age: 26.39 years old) and 31 healthy controls (mean age: 25.71 years old) and investigated the association between EC and stereoacuity. In addition, we explored EC changes after perceptual learning in a novel longitudinal dataset including 9 patients with amblyopia (mean age: 15.78 years old). We found consistent evidence from the two datasets indicating that the aberrant EC from V2v to LO2 is crucial for the stereoscopic deficits in the patients with amblyopia: it was weaker in the patients than in the controls, showed a positive linear relationship with the stereoscopic function, and increased after perceptual learning in the patients. In addition, higher-level dorsal (V3d, V3A, and V3B) and ventral areas (LO1 and LO2) were important nodes in the network of abnormal ECs associated with stereoscopic deficits in the patients with amblyopia. Our research provides insights into the neural mechanism underlying stereopsis deficits in patients with amblyopia and provides candidate targets for focused stimulus interventions to enhance the efficacy of clinical treatment for the improvement of stereopsis deficiency.

## Highlights

• Abnormal effective connectivity inherent stereopsis defects in amblyopia was studied.
• A weakened connection from V2v to LO2 relates to stereopsis defects in amblyopia.
• Higher-order visual cortices may serve as key nodes to the stereopsis defects.
• An independent longitudinal dataset was used to validate the obtained results.

## 1 Introduction

Amblyopia is the most common cause of abnormal binocular vision, with a prevalence of 1.44% ∼ 4.3% (Fu et al., 2019, Mostafaie et al., 2020), and is considered to be derived from abnormal cortical development caused by abnormal visual experience in early life. Stereopsis is the most advanced binocular function to obtain depth perception based on binocular disparity cues (DeAngelis, 2000). Abnormal stereoscopic vision is common in patients with amblyopia. This deficit may remain even after traditional treatments such as refractive correction and patching (Levi et al., 2015). Stereoscopic defects could destroy the depth perception and visually guided hand-eye coordination of patients with amblyopia, which affect their daily life and limit their career choices (Levi et al., 2015). Therefore, new treatment strategies targeting stereopsis restoration have garnered interest in recent years. Perceptual learning is a candidate method to improve the stereopsis of patients with amblyopia (Kraus and Culican, 2018, Levi et al., 2015, Portela-Camino et al., 2018, Xi et al., 2014). However, perceptual learning is highly repetitive, boring, and time consuming and thus has high requirements for patient compliance (Levi, 2020). As a result, it is often conducted in a laboratory-based setting and is difficult to apply to daily clinical practice. The cortical mechanism of stereo vision defects and recovery in patients with amblyopia remains unclear, which hinders the prediction of individual response to treatment and the development of new therapies.

Although the neural mechanism of stereopsis deficits in patients with amblyopia has not yet been elucidated, the processing mechanism of stereopsis has been extensively studied in populations with normal binocular development. The perception of stereopsis depends on binocular disparity cues, which refer to the subtle differences between the corresponding images of two retinas (Tyler, 1990). An increasing number of studies agree that both the dorsal and ventral visual pathways play crucial roles in stereoscopic processing with different specifications and can interact at multiple levels (Chandrasekaran et al., 2007, Iwaki et al., 2011, Janssen et al., 2018, Nelissen et al., 2009, Neri, 2005, Preston et al., 2008, Welchman et al., 2005). First, early experiments proved that disparity-selective neurons existed in multiple cortical areas of the dorsal and ventral streams, including the V1, V2, V3, V3A, V3B, V4, middle temporal area (MT/V5), medial superior temporal area (MST), lateral occipital cortex (LOC), and intraparietal sulcus (IPS) (Andersen et al., 1995, DeAngelis, 2000, Hubel and Wiesel, 1970, Joly and FrankÃ, 2014, Poggio et al., 1988). These disparity-selective neurons constituted the physiological basis of disparity-defined stereopsis perception. Second, human functional magnetic resonance imaging (fMRI) studies reported that neural activation in the visual cortex covaried with stereoscopic perception within the detectable disparity range (Backus et al., 2001), which suggested a correlation between the fMRI activation response and behavioral performance. This evidence supports fMRI as an effective noninvasive method for studying the neural mechanism of human stereoscopic processing. In addition, the functional characteristics exhibited by brain regions are not inherent to the regions themselves but result from a specific set of interactions within the integrated networks in which they are embedded (Backus et al., 2001, Hutchison and Gallivan, 2018). In brief, stereoscopic processing may involve complex interactions between multiple regions in the dorsal and ventral visual pathways. However, whether and how the interactions of the two visual pathways are aberrant in patients with amblyopia is still unclear.

Previous neuroimaging studies have reported abnormal functional connectivity within the early visual network (Mendola et al., 2018), aberrant functional connectivity between the primary and higher-level visual networks (Dai et al., 2019, Ding et al., 2013), and abnormal structural connectivity in the ventral and dorsal visual streams (Duan et al., 2015, Li et al., 2015, Tsai et al., 2019), suggesting disrupted functional and structural interactions in patients with amblyopia. One study also reported that functional connectivity in the lingual gyrus was correlated with stereoacuity (Liang et al., 2017). Nevertheless, functional connectivity only reflects the correlation of activity between spatially remote brain regions, which can be caused by many factors, including direct influence, indirect influence, and shared influence (Fingelkurts et al., 2005). Therefore, the results of the functional connectivity analysis must be interpreted with caution. Instead, effective connectivity (EC) indicates the causal influence one brain region exerts over another (Stephan and Friston, 2010) and is informative for feedforward and feedback information, which is of great significance to the integration of visual information (Lamme et al., 1998, Premereur et al., 2015, Wu et al., 2010, Youssofzadeh et al., 2015), especially for stereoscopic perception, as it involves complex interactions of multiple brain regions. The spectral dynamic causal modeling (DCM) method was developed specifically for modeling resting-state fMRI data and provided a computationally efficient way to estimate EC from fitting cross spectra by combining hemodynamic and neural-dynamic information (Friston et al., 2014, Park et al., 2018, Zeidman et al., 2019a, Zeidman et al., 2019b). On the other hand, the parameter estimate of a connection in the spectral DCM frame can reflect the excitatory or inhibitory influence of one brain region on another at the population level (Bastos et al., 2012, Zeidman et al., 2019a). Evidence from rodents pointed out that the balance between excitation and inhibition was disrupted during the development of patients with amblyopia (Turrigiano et al., 2002, Zhou et al., 2017) and excessive cortical inhibition may cause the deterioration of spatial visual ability (Baroncelli et al., 2011, Wong et al., 2005). These findings suggest that information about the excitatory and inhibitory influence may also contribute to understanding the neural mechanism of stereoscopic deficits in patients with amblyopia. Two studies (Dai et al., 2021, Li et al., 2011) reported abnormal EC in patients with amblyopia. However, they did not investigate how these EC abnormalities were related to stereopsis.

Based on this background, our study aimed to explore abnormalities in EC between visual brain regions underlying stereopsis defects in patients with amblyopia. The spectral DCM method was employed in a cross-sectional dataset to compare group differences in EC differences between patients with amblyopia and healthy controls and the relationship between these abnormal ECs and stereopsis defects. To validate the results found in the cross-sectional dataset, we also included an independent longitudinal dataset to investigate the relationship between EC changes and stereoacuity improvement after perceptual learning treatment in the patients. We predicted that patients with amblyopia exhibited a wide range of abnormal ECs within and between the dorsal and ventral visual pathways, including feedforward and feedback (Li et al., 2011) and these abnormalities may be associated with stereoscopic defects in patients. We included regions of interest (ROIs) associated with stereoscopic processing according to previous studies, which could provide more targeted information for stereopsis function. EC analysis could provide more comprehensive information for the direction of feedforward and feedback. More targeted and comprehensive information may help to establish a link between EC and stereopsis function. In addition, we also predicted that ECs associated with stereopsis defects found in the cross-sectional dataset may change with the improvement of stereopsis after perceptual learning treatment in the longitudinal dataset.

## 2 Materials and methods

### 2.1 Participants and clinical measurements

**Fig. 1:**
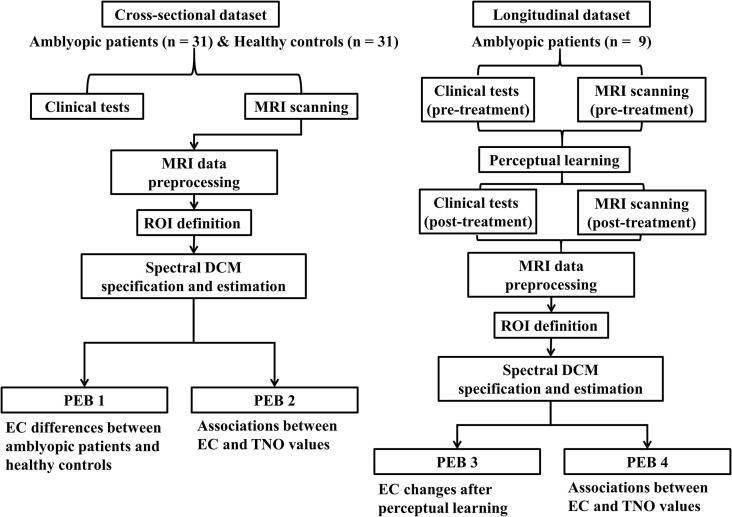
Flowchart of the current study. Abbreviations: MRI: magnetic resonance imaging; ROI: region of interest; DCM: dynamic causal modeling; PEB: parametric empirical Bayes; EC: effective connectivity; TNO: the Netherlands Organization for applied scientific research, referring to the TNO stereo test here.

This study was approved by the ethics committee of West China Hospital of Sichuan University for human studies and registered in the Chinese Clinical Trial Registry (registration number: ChiCTR2000040912). Written informed consent was obtained from each subject before participation. This study consisted of two independent datasets. The cross-sectional dataset included 31 patients with amblyopia and 31 healthy controls (HCs). The longitudinal dataset included 9 patients with amblyopia who underwent two MRI scan sessions before and after stereoscopic perceptual learning treatment. The flowchart of the current study (Fig. 1) and detailed information on the participants are presented below.

#### 2.1.1 Cross-sectional dataset

Patients with amblyopia were consecutively recruited between September 2020 and September 2021 from the Department of Ophthalmology, West China Hospital, Sichuan University (Chengdu, China). All participants were subjected to a comprehensive ophthalmological and orthoptic examination to confirm whether they met the criteria of our study. Examinations included visual acuity, TNO (the Netherlands Organization for applied scientific research) stereo test (Lameris Intrumenten, Groenekan, the Netherlands, 9th or 10th edition https://www.ootech.nl/), objective refraction assessment, fundus examination, eye alignment (cover test), and eye movements. The inclusion criteria for patients with amblyopia were as follows: 1) amblyopic patients with anisometropia, strabismus, or both; 2) age between 18 and 40 years old; 3) right-handed; and 4) best-corrected visual acuity (BCVA) of the amblyopic eye worse than 0.1 logMAR or an interocular BCVA difference of more than two lines. All the HCs recruited met the following criteria: 1) age between 18 and 40 years old; 2) right-handed; 3) BCVA not worse than 0 logMAR in either eye; 4) normal binocular visual function, and 5) no history of amblyopia or strabismus. Patients and controls were excluded if they had 1) any organic eye disease; 2) a history of head trauma or other psychiatric or neurological disorders; 3) metabolic diseases such as diabetes and hyperthyroidism; or 4) any contraindications to MRI measurement.

#### 2.1.2 Longitudinal dataset

The longitudinal dataset included a separate group of patients with amblyopia who were recruited between 2014 and 2016 in the same department as mentioned above. The inclusion criteria were as follows: 1) right-handed; 2) older than 12 years old; 3) a clear diagnosis of amblyopia; and 4) able to understand and perform training tasks. The exclusion criteria were as follows: 1) any organic eye disease; 2) a history of head trauma or other psychiatric or neurological disorders; 3) metabolic diseases such as diabetes and hyperthyroidism; 4) any MRI contraindications.

For all patients included, we adopted the training paradigm reported in the previous literature (Chopin et al., 2021, Ding and Levi, 2011). All patients received perceptual learning with the task of identifying cross-disparity and uncross-disparity under the condition of balanced interocular contrast. Each session of training contained 500 disparity judgment trials, of which 250 crossed and uncrossed disparities each appeared randomly. Each session was randomly divided into four blocks of 100 trails. Patients could choose to rest for minutes after every block. Considering the interindividual differences in the therapeutic responses reported by previous studies (Ding and Levi, 2011, Fendick and Westheimer, 1983, Gantz et al., 2007, Portela-Camino et al., 2021), we set a minimum of 20 sessions (10000 trials) to ensure the training effect and adopted flexible training sessions to maximize stereo improvement for each patient. Variable training sessions for every training day were also adopted to ensure that patients remained focused on training tasks during perceptual learning, as the effect of perceptual learning may be influenced by patients’ attention (Liu & Zhang, 2019). Therefore, for each training day, 2 to 4 sessions of training were given to a patient depending on his/her level of fatigue. Finally, the total training duration was varied from 20 to 49 sessions in a range of 1 to 2 weeks for each patient to finish all the training sessions.

### 2.2 MR imaging acquisition

#### 2.2.1 Cross-sectional dataset

All participants underwent an MRI examination using a 3.0 T system (Tim Trio; Siemens Health ineers, Erlangen, Germany) equipped with a 32-channel phased-array head coil. Participants were instructed to keep their eyes closed and not fall asleep during the acquisition. Earplugs and foam pads were used to reduce scanning noise and minimize head motion. A three-dimensional T1-weighted image was acquired using a spoiled gradient-recalled echo sequence with the following parameters: repetition time (TR) = 2400 ms; echo time (TE) = 2.01 ms; flip angle = 8°; matrix size = 320 × 320; field of view = 256 × 256 mm^2^; slice thickness = 0.8 mm; and voxel size = 0.8 × 0.8 × 0.8 mm^3^. The acquisition time for the T1-weighted image was approximately 7 min. A gradient-echo echo-planar imaging sequence was used to obtain blood oxygen level-dependent sensitive MR images with the following parameters: TR = 700 ms; TE = 37.8 ms; flip angle = 52°; slice thickness = 2.1 mm without intersection gaps; matrix size = 100 × 100; field of view = 210 × 210 mm^2^; and voxel size = 2.10 × 2.10 × 2.10 mm^3^; multiband accelerator factor = 8. Each subject continuously underwent two sessions of functional scanning with opposite phase encoding directions (from right to left and vice versa), and each session contained 415 time points, resulting in a total imaging time of 9 min 41 sec. An experienced neuroradiologist (W. X.) checked the structural and functional image quality.

#### 2.2.2 Longitudinal dataset

For the longitudinal dataset, we performed the first MRI scan before treatment. After finishing all training procedures, patients underwent a second MRI scan. The second MRI scanning was no more than two days after the end of training. MRI images of the longitudinal dataset were acquired using the same MRI scanner as mentioned above equipped with a 12-channel phased-array head coil. Participants were instructed to keep their eyes closed and not fall asleep during the acquisition. Earplugs and foam pads were used to reduce scanning noise and minimize head motion. Three-dimensional T1-weighted images were acquired with the following parameters: TR = 2250 ms; TE = 2.62 ms; flip angle = 9°; matrix size = 256 × 256; slice thickness = 1 mm without intersectional gaps; voxel size = 1.0 × 1.0 × 1.0 mm^3^. The acquisition time for the T1-weighted image was approximately 5 min 30 sec. Resting-state functional images were obtained using an echo-planar imaging sequence with the following parameters: TR = 2000 ms; TE = 35 ms; flip angle = 68°; matrix size = 64 × 64; slice thickness = 3 mm with 20% intersectional gap; and voxel size = 3.25 × 3.25 × 3.60 mm^3^. The phase encoding direction was from front to posterior. Each session contained 220 time points, resulting in a total imaging time of 7 min 20 sec.

### 2.3 MR imaging preprocessing

#### 2.3.1 Cross-sectional dataset

First, the MRI Quality Control Tool MRIQC (Esteban et al., 2017) version 0.16.1 was used to further check the image quality of T1 and fMRI data in the cross-sectional dataset. No functional MRI data were excluded from further analysis due to excessive head motion (i.e. mean framewise displacement > 0.5 mm). Then, the first ten volumes of each blood oxygen level-dependent (BOLD) session were discarded, and the next preprocessing steps were performed using fMRIPrep version 20.2.1 (Esteban et al., 2019), which is based on Nipype 1.5.1 (Gorgolewski et al., 2011). A full description of the preprocessing pipeline using fMRIprep can be found in the Supplementary Materials. Finally, fMRI data of two opposite phase encoding directions were concatenated, resulting in 810 volumes for each subject in the following analysis, and the preprocessed images were smoothed with a 6 mm full width at half maximum Gaussian kernel.

#### 2.3.2 Longitudinal dataset

For the longitudinal dataset, MRI image preprocessing was performed using Statistical Parametric Mapping software (SPM12) (http://www.fil.ioon.ucl.ac.uk/spm/) with the following steps: eliminating the first ten volumes, slice timing correction, motion correction, structural and functional image coregistration, segmentation, normalization, and smoothing using a kernel with a full-width half-maximum of 6 mm. No subject was excluded due to excessive head motion (translational motion > +1 mm or rotational movement > +1 degree) during functional MRI scanning.

### 2.4 Methods common for both datasets

The following analysis methods were common in both datasets. All analyses of spectral DCM and parametric empirical Bayes (PEB) were conducted with SPM12 (https://www.fil.ion.ucl.ac.uk/spm/) using codes based on MATLAB 2018b, following the guidance of previous studies (Zeidman et al., 2019a, Zeidman et al., 2019b). We only provided the main details of our analysis steps here. More information can be found in the Supplementary Materials.

#### 2.4.1 ROI definition

**Fig. 2:**
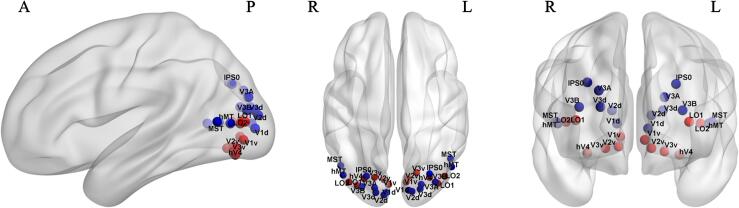
Visualization of ROIs in the dorsal and ventral visual streams. Blue spheres code dorsal visual regions, whereas red spheres code ventral visual areas. Abbreviations: ROIs: regions of interest; V1: primary visual cortex; V2: secondary visual cortex; V3: visual area V3; hV4: human visual region V4; hMT: human middle temporal region; MST: medial superior temporal area; IPS: intraparietal sulcus; LO: lateral occipital; v: ventral; d: dorsal; A: anterior; P: posterior; R: right; L: left. (For interpretation of the references to colour in this figure legend, the reader is referred to the web version of this article.)

First, a total of 14 ROIs were defined to represent stereopsis related cortical regions in the dorsal and ventral visual pathways, including 8 dorsal ROIs (V1d, V2d, V3d, V3A, V3B, hMT, MST, IPS0) and 6 ventral ROIs (V1v, V2v, V3v, hV4, LO1, LO2) (Fig. 2). The ROIs were defined utilizing the voxel-based probabilistic atlas (Wang et al., 2015), which was created in Montreal Neurological Institute (MNI) space and frequently used in previous studies on the visual system (Cichy et al., 2016a, Cichy et al., 2016b, Hindy et al., 2016, Mackey et al., 2017). To minimize bias of variations in ROI size on the estimates of connectivity and signal-to-noise ratio differences, the ROIs were arbitrarily constricted to a set of spheres with a radius of 3 mm and positioned at the center of each corresponding single brain region defined by a full probability map (Wang et al., 2015) as in a previous study (Backner et al., 2020). We set this specific size to ensure that all the ROIs remain in the defined brain regions and do not overlap with each other. Corresponding regions in both hemispheres were combined and defined as one bilateral ROI. The detailed coordinate information is listed in the Supplementary Materials, Table S1.

#### 2.4.2 Time series extraction

Then, BOLD fMRI time series corresponding to the aforementioned 14 ROIs were extracted from the preprocessed data to establish the residuals of a general linear model. Six head motion parameters and white matter/cerebrospinal fluid signals were added to the model as nuisance regressors.

#### 2.4.3 First-level DCM specification and estimation

After extracting the time series values of all ROIs, we specified a fully connected DCM model (each node connects to itself and all other nodes) consisting of 14 ROIs for each subject. Then, subject-level model estimation based on standard variational Bayes procedures (variational Laplace) under the frequency domain was performed.

#### 2.4.4 Second-level PEB analysis

After participant-level DCM models were specified and estimated, the group-level PEB model using Bayesian posterior inference was performed. In the current study, four independent group-level PEBs were conducted (Fig. 1). PEB 1 and PEB 2 were applied to the cross-sectional dataset, whereas PEB 3 and PEB 4 were applied to the longitudinal dataset. The basic principles of PEB matrix design were as follows: the first column is a constant term, modeling group means, the second column is the covariate of interest, and subsequent columns encode other covariates of no interest (mean centred), i.e., age, sex, and years of education in our study (Friston et al., 2016). Specifically, PEB 1 was designed for group comparison of EC between patients with amblyopia and controls. The design matrix of PEB1 was encoded as group means, group differences, education years, age, and sex. PEB 2 was designed to explore the linear relationship between EC and TNO values in the cross-sectional dataset. The design matrix was designed in the following sequence: group means, TNO values, group, education years, age, and sex. PEB 3 was designed to investigate the EC changes after perceptual learning, with a design matrix encoded in the order of group means, group differences, education years, age, and sex. PEB 4 was designed to investigate the linear relationship between EC and TNO values in the longitudinal dataset. The design matrix was designed in the following sequence: group means, TNO values, group, education years, age, and sex.

Then, exploratory Bayesian model reduction was performed to optimize the full PEB group-level model by removing one or more connectivity parameters and deriving the model evidence (free energy). Finally, a Bayesian model average was performed to average the connectivity parameters of the best models, weighted by their evidence. In the results section, we reported the EC parameter estimates of this average over the best models.

### 2.5 Statistical analysis of clinical data

SPSS software (version 23.0; SPSS, Inc.) was used for the statistical analyses of demographical and clinical data. Participants who had no measureable stereopsis were assigned a stereoacuity of 5000 arcsec, and stereoacuity scores of each participant were log10 transformed to meet the normality assumption. Additionally, the BCVA values were converted to logMAR. Independent sample t-tests were used to compare the measures of the amblyopia group and HC group, while paired sample t-tests were used to compare the parameters between pre- and post-treatment amblyopia. Chi-square tests were used for categorical variables (gender) of group differences.

## 3 Results

### 3.1 Demographics and clinical measurements of the two datasets

Table 1 summarizes the demographics and clinical measurements of the two datasets. For the cross-sectional dataset, a total of 31 adult patients with amblyopia (age between 18 and 37 years old, mean age: 26.39 ± 4.94 years; male/female: 10/21) and 31 HCs (mean age: 25.71 ± 2.75 years old; male/female: 8/23) were included. The patients and controls were similar in age (*p* = 0.51) and gender (*p* = 0.58) but differed significantly in education years (*p* < 0.001). Patients with amblyopia had significantly higher BCVA (*p* < 0.001) and TNO values (*p* < 0.001), which represent worse visual acuity and worse stereo acuity.

**Table 1:** Demographics and clinical measurements of the cross-sectional dataset and the longitudinal dataset.

Cross-sectional dataset				
	Amblyopia (n = 31)	HC (n = 31)	T value/chi-square	P-value
Age (years)	26.39 ± 4.94	25.71 ± 2.75	0.668	0.51
Gender (M/F)	N = 10/21	N = 8/23	0.313	0.58
Education (years)	15.55 ± 2.05	17.87 ± 2.00	−4.524	< 0.001
BCVA (logMAR)	0.70 ± 0.47	−0.02 ± 0.04	8.538	< 0.001
TNO (log_10_)	3.60 ± 0.34	1.78 ± 0.13	27.575	< 0.001

Longitudinal dataset				
	Pre-treatment (n = 9)	Post-treatment (n = 9)	T value/chi-square	P-value

Age (years)	15.78 ± 3.15		–	–
Gender (M/F)	N = 3/6		–	–
Education (years)	9.67 ± 3.08		–	–
BCVA (logMAR)	0.43 ± 0.16	0.24 ± 0.12	5.128	0.01
TNO (log_10_)	2.98 ± 0.87	2.05 ± 0.32	4.323	< 0.01

The mean ± standard deviation of age, education years, BCVA, and TNO values for patients with amblyopia and healthy controls for both datasets are presented in the table. Abbreviations: HC: healthy control; M: male; F: female; BCVA: best-corrected visual acuity; TNO: the Netherlands Organization for applied scientific research, referring to the TNO stereo test here.

For the longitudinal dataset, a total of 9 patients with amblyopia (age between 12 and 23 years old, mean age: 15.78 ± 3.15 years; male/female: 3/6) were included. Patients showed significantly lower BCVA (*p* = 0.01) and TNO values (*p* < 0.01) after perceptual learning treatment, which represent improvements in visual acuity and stereo acuity.

### 3.2 DCM results for the cross-sectional dataset

**Fig. 3:**
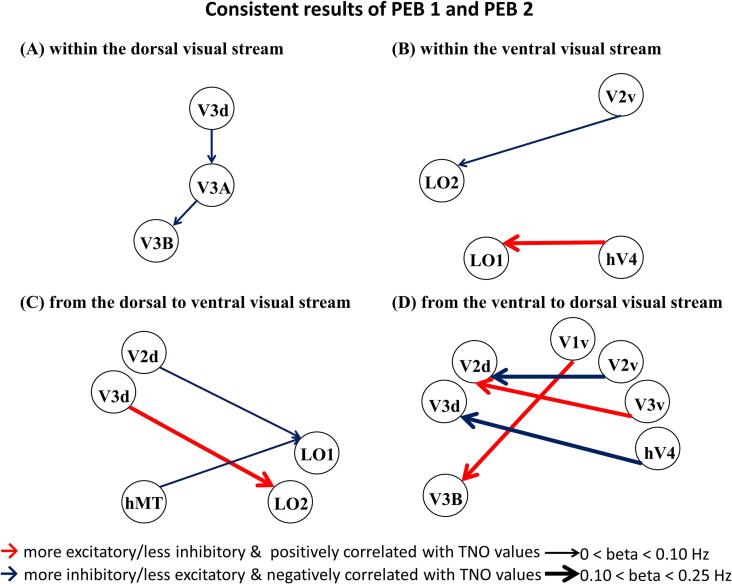
Consistent results of PEB 1 and PEB 2 based on the cross-sectional dataset (Pp > 0.99). This figure presents connections that show group differences (amblyopia minus control) and have a linear relationship with TNO values. Only consistent results of PEB1 and PEB 2 with very strong evidence (Pp > 0.99) are depicted. Lines with arrows represent connections (A) within the dorsal visual stream; (B) within the ventral visual stream; (C) from the dorsal to ventral visual stream; and (D) from the ventral to dorsal visual stream. The arrows indicate the direction of connections. Red lines denote connections that are stronger in patients with amblyopia and have a positive linear relationship with TNO values, while blue lines denote connections that are weaker in patients with amblyopia and have a negative linear relationship with TNO values. Lines are scaled by the effect size of PEB 1 from 0 to 0.25 Hz. Abbreviations: PEB: parametric empirical Bayes; Pp: posterior probability; TNO: the Netherlands Organization for applied scientific research, referring to the TNO stereo test here. (For interpretation of the references to colour in this figure legend, the reader is referred to the web version of this article.)

The detailed results of group differences (results of PEB 1, amblyopia minus control) and linear relationships between EC and TNO values (results of PEB 2) based on the cross-sectional dataset can be found in the Supplementary Materials (Fig. S1 and Fig. S3). Similar results were obtained by the PEBs with and without nuisance regressors (Supplementary Materials, Fig. S2, and Fig. S4). Here, we focused on the consistent results of PEB 1 (with education years, age, and sex as covariates of no interest) and PEB 2 (with the group, education years, age, and sex as covariates of no interest). Only ECs showing “very strong evidence” (Pp > 0.99) are illustrated in Fig. 3. We presented our results in four categories based on the source and target regions: within the dorsal visual pathway, within the ventral visual pathway, from the dorsal to ventral visual pathway, and from the ventral to dorsal visual pathway.

#### 3.2.1 Within the dorsal and ventral visual pathways

The upper panels of Fig. 3 depict ECs within the dorsal (left) and ventral (right) visual pathways that showed group differences (patients vs. controls) and had a linear relationship with the TNO values. Within the dorsal visual pathway, two ECs (from V3d to V3A and from V3A to V3B) were weaker in patients with amblyopia than in HCs and had negative linear relationships with the TNO values; that is, more inhibitory/less excitatory influence of these ECs was associated with worse stereo acuity in patients with amblyopia.

Within the ventral visual pathway, the EC (from V2v to LO2) was lower in patients with amblyopia than in HCs and had a negative linear relationship with the TNO values. In addition, the EC from hV4 to LO1 was stronger in patients with amblyopia than in HCs and had a positive linear relationship with the TNO values; that is, a greater excitatory/less inhibitory influence of this EC was associated with worse stereopsis function in patients with amblyopia.

#### 3.2.2 Between dorsal and ventral visual pathways

The bottom panels of Fig. 3 depict ECs between dorsal and ventral visual pathways that showed group differences (patients vs. controls) and had a linear relationship with TNO values. Compared with HCs, patients presented lower connectivities from the dorsal to the ventral visual pathway (from V2d to LO1 and from hMT to LO1) and from the ventral to the dorsal visual pathway (from V1v to V3B and from V3v to V2d), which also showed negative linear relationships with TNO values. In addition, higher connectivities were observed in patients than in HCs from the dorsal to the ventral visual pathway (from V3d to LO2) and from the ventral to the dorsal visual pathway (from V1v to V3B and from V3v to V2d), which also showed positive linear relationships with TNO values.

### 3.3 DCM results for the longitudinal dataset

**Fig. 4:**
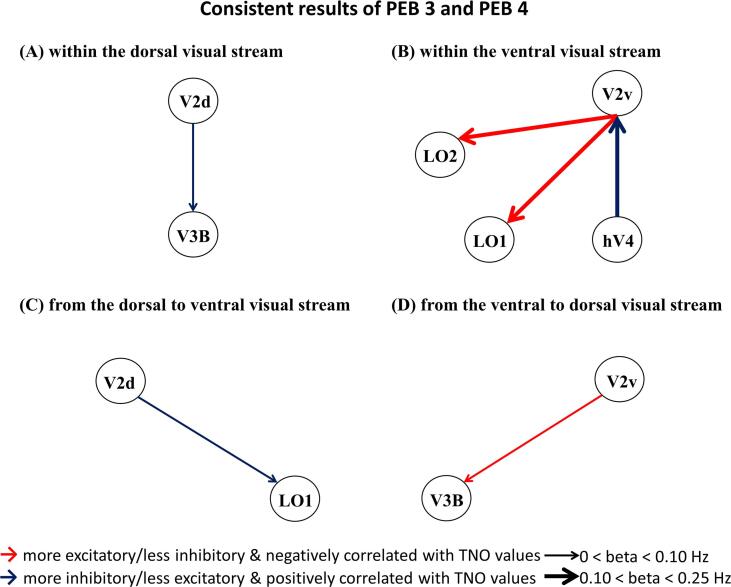
Consistent results of PEB 3 and PEB 4 based on the longitudinal dataset (Pp > 0.99). This figure presents connections that show group differences (post-treatment minus pre-treatment) and have a linear relationship with TNO values. Only consistent results of PEB 3 and PEB 4 with very strong evidence (Pp > 0.99) are depicted. Lines with arrows represent connections (A) within the dorsal visual stream; (B) within the ventral visual stream; (C) from the dorsal to ventral visual stream; and (D) from the ventral to dorsal visual stream. The arrows indicate the direction of connections. Red lines denote connections that are increased after treatment and have a negative linear relationship with TNO values; while blue lines denote connections that are decreased after treatment and have a positive linear relationship with TNO values. Lines are scaled by the effect size of PEB 3 from 0 to 0.25 Hz. Abbreviations: PEB: parametric empirical Bayes; Pp: posterior probability; TNO: the Netherlands Organization for applied scientific research, referring to the TNO stereo test here. (For interpretation of the references to colour in this figure legend, the reader is referred to the web version of this article.)

The detailed results of group differences (results of PEB 3, post-treatment minus pre-treatment) and linear relationships between ECs and TNO values (results of PEB 4) based on the longitudinal dataset can be found in the Supplementary Materials (Fig. S5 and Fig. S7). Similar results were obtained by the PEBs with and without nuisance regressors (Supplementary Materials, Fig. S6, and Fig. S8). Here, we focused on the consistent results of PEB 3 (with education years, age, and sex as covariates of no interest) and PEB 4 (with the group, education years, age, and sex as covariates of no interest). Only ECs showing “very strong evidence” (Pp > 0.99) are illustrated in Fig. 4.

#### 3.3.1 Within the dorsal and ventral visual pathways

The upper panels of Fig. 4 depict ECs within the dorsal (left) and ventral (right) visual pathways that showed group differences (post-treatment vs. pre-treatment) which also had a linear relationship with TNO values. Within the dorsal visual pathway, the EC from V2d to V3B decreased after treatment which also presented a positive linear relationship with the TNO values in the patients; that is, more inhibitory/less excitatory influence of these ECs were associated with better stereo acuity in patients with amblyopia after treatment.

Within the ventral visual pathway, the EC from hV4 to V2v was decreased after treatment which also showed a negative linear relationship with the TNO values. In addition, two ECs (from V2v to LO1 and LO2) increased after treatment in patients with amblyopia and had a negative linear relationship with TNO values; that is, a greater excitatory/less inhibitory influence of these ECs was associated with better stereopsis function in post-treatment amblyopia.

#### 3.3.2 Between the dorsal and ventral visual pathways

The bottom panels of Fig. 4 depict ECs between dorsal and ventral visual pathways that showed group differences (post-treatment vs. pre-treatment) and presented a linear relationship with TNO values simultaneously. The EC from V2d to LO1 decreased in post-treatment patients and had positive linear relationships with TNO values. In addition, the EC from V2v to V3B increased after treatment in the patients and had negative linear relationships with TNO values.

### 3.4 Major DCM results

We observed the most consistent finding across the two independent datasets: the EC from V2v to LO2, which showed a reliably weaker EC in patients with amblyopia in the cross-sectional dataset (Fig. 3), increased connectivity after treatment in the longitudinal dataset (Fig. 4) and had reliable negative linear relationships with TNO values in both datasets (see Fig. 3 and Fig. 4).

## 4 Discussion

By adopting DCM and PEB analysis methods for resting-state fMRI data, the current study investigated EC abnormalities in patients with amblyopia and explored the relationship between these abnormal ECs and stereopsis function in a cross-sectional dataset. To verify the results of the cross-sectional dataset, we also investigated EC changes after perceptual learning treatment in patients with amblyopia and explored the association between these altered ECs and stereo vision in an independent longitudinal dataset. The results showed widespread abnormal ECs within and between the dorsal and ventral visual pathways in the patients with amblyopia and these abnormalities were associated with stereopsis deficits. Specifically, higher-level dorsal (V3d, V3A, and V3B) and ventral visual regions (LO1 and LO2) played key roles in stereoscopic processing defects in patients with amblyopia. Notably, convergent evidence from our cross-sectional and longitudinal datasets suggested that the EC from V2v to LO2 played a crucial role in stereoscopic defects in patients with amblyopia. The evidence includes that the EC from V2v to LO2 was abnormally weaker in patients with amblyopia, showed a positive association with stereopsis function, and was increased after perceptual learning treatment in the patients. This study shed light on the possible neural substrate of stereopsis defects in patients with amblyopia and identified potential targets for efficacy prediction and intervention in stereopsis recovery.

### 4.1 Abnormal EC from V2v to LO2 associated with stereopsis defects

The most robust result related to stereoscopic defects in patients with amblyopia was the EC from V2v to LO2, which was weaker in patients with amblyopia and increased after perceptual learning therapy. This consistent evidence from cross-sectional and longitudinal datasets provided strong evidence that the EC from V2v to LO2 played a significant role in stereopsis function. V2v belongs to the early ventral visual cortex and is located in the lingual gyrus. LO2 belongs to the higher-level ventral visual cortex and is the subregion of the LOC (Larsson and Heeger, 2006). Previous studies have reported decreased cortical thickness of the V2/lingual gyrus (Du et al., 2009, Liang et al., 2019, Qi et al., 2016) as well as a reduced volume of the LOC in patients with amblyopia (Lu et al., 2019). There have also been studies reporting abnormal connectivity of the ventral visual stream both in structure (Tsai et al., 2019) and in function (Dai et al., 2019). Only one study reported an association between stereovision and functional connectivity in the lingual gyrus of patients with amblyopia (Liang et al., 2017). Our study confirms abnormal connectivity in the ventral visual stream of patients with amblyopia and further suggests the direction of this abnormality. Furthermore, our results suggest a reliable association between an abnormal EC (from V2v to LO2) in the ventral visual stream and stereopsis deficits in patients with amblyopia. Evidence from populations with normal binocular vision development has demonstrated the important roles of the ventral visual areas V2v and LO2 in stereoscopic processing: V2 is the first visual area to realize binocular correspondence (Chen et al., 2017), which is the first step in stereoscopic perception, while LO2 has high accuracy for the discrimination of crossed and uncrossed disparities (Li et al., 2017, Preston et al., 2008). These findings provide support for our results. In general, the ventral visual stream processes high spatial frequency, static information to represent fine and global stereopsis suitable for the processing of complex random-dot stereograms and recognizing objects (Tyler, 1990). The relationship between stereoacuity deficiency and abnormal EC within the ventral visual stream we found here may underlie defective fine and global stereopsis processing in patients with amblyopia.

Moreover, the results of the longitudinal dataset demonstrated that the EC from V2v to LO2 increased after perceptual learning treatment in patients with amblyopia. The EC changes observed in patients with amblyopia after perceptual learning may reflect the neural plasticity of the visual cortex (Basgoze et al., 2018, Tailor et al., 2017), suggesting that perceptual learning could modify the treatment-related brain area, and the observed changes were not limited to one cortical area (Dosher & Lu, 2017) because the brain is an interconnected network. Therefore, the substrates of perceptual learning are more complex than the early claims of plasticity in V1 (Karni & Sagi, 1991). Instead, reweighting neuronal responses from one area to another (e.g., from V2v to LO2) could account for the stereopsis improvements observed in patients with amblyopia. Zhai and his colleagues (2013) reported significantly increased activation via the amblyopic eye in the V1, V2, V3, bilateral temporal lobes, and right cingulate gyrus after perceptual learning treatment, indicating the positive effect of perceptual learning on the local function of the occipital visual cortex and temporal cortex (Zhai et al., 2013). Our study confirms the positive impact of perceptual learning on the visual cortex and further suggests potential EC alterations underlying stereopsis improvement. In addition to perceptual learning, recent studies also reported that the noninvasive focus stimulus technique was another potential strategy to restore stereopsis function for adults with amblyopia (Castano-Castano et al., 2019, Hess et al., 2014, Tuna et al., 2020), which could induce or manipulate the underlying excitability, connectivity, and plasticity of the human brain (Ferreri & Rossini, 2013). In future studies, combining perceptual learning with the focus stimulus technique may contribute to the understanding of the mechanism of stereopsis recovery in patients with amblyopia and promote the effectiveness of treatment. Our findings suggest that EC from V2v to LO2 could be a candidate therapeutic process to be enhanced or promoted with mechanistically focused intervention approaches thus promoting the improvement of stereoscopic perception in patients with amblyopia.

### 4.2 Abnormal ECs within and between the dorsal and ventral visual stream associated with stereopsis defects

In addition to the abnormal interactions within the ventral visual streams, anomalous ECs within the dorsal visual pathway as well as between the two pathways were also found to be associated with stereoacuity in our cross-sectional dataset. Within the dorsal stream, we found that ECs from V3d to V3A and from V3A to V3B were weaker in patients with amblyopia and positively associated with stereoacuity. Previous studies have reported disturbed structural connectivity (Li et al., 2015) and functional connectivity (Ding et al., 2013) in the dorsal visual stream of patients with amblyopia. Here, our study further suggested that the direction of abnormal connectivity was feedforward and emphasized the crucial contributions of V3d, V3A, and V3B to stereoscopic processing, which was consistent with previous evidence (Henderson et al., 2019, Ip et al., 2014, Li et al., 2017, Tyler et al., 2006). Specifically, V3d was proven to be crucial in the early transformation of binocular disparity to depth perception due to its sensitivity to crossed and uncrossed disparities (Li et al., 2017) as well as changes in interocular disparity correlation (Ip et al., 2014). In addition, evidence from 7 T fMRI reported that human V3A and V3B regions contain selective and organized structures supporting stereoscopic processing (Goncalves et al., 2015). V3A was regarded as the most robust dorsal region for depth coding (Henderson et al., 2019), as it was highly sensitive to binocular disparity (Backus et al., 2001, Berryhill, 2009, Tsao et al., 2003, Welchman, 2016) and could influence both the minimum and maximum detectable disparities (Chen et al., 2020). It also participated in integrating cues for 3D shape perception (Welchman et al., 2005), decision-related recognition activities in disparity discrimination tasks (Cottereau et al., 2014), and guiding hand movements (e.g., grasping) (Cottereau et al., 2012). In contrast, V3B was thought of as the main center of the depth representation (Tyler et al., 2006) because of its crucial role in integrating various cues for depth perception, including disparity and motion cues (Ban et al., 2012), texture and disparity cues (Murphy et al., 2013), and monocular and binocular cues (Sun et al., 2016). On the whole, the dorsal stream processes motion and transient information to represent coarse, local stereopsis suitable for stereo movement processing and promote visually guided actions (Tyler, 1990). The association between worse stereoacuity and abnormal ECs within the dorsal visual stream reported here may underlie coarse stereopsis perception deficits.

In addition, we also found that abnormal interactions between the dorsal and ventral visual pathways were associated with stereopsis in our cross-sectional dataset. Previous studies have reported disrupted vertical occipital fasciculus, which suggested affected communication between the dorsal and ventral visual streams in patients with amblyopia (Duan et al., 2015). To achieve stereoscopic perception, dorsal and ventral visual streams could interact at multiple levels (Chandrasekaran et al., 2007, Preston et al., 2008), which were not only reflected in functional (Iwaki et al., 2011, Nelissen et al., 2009) but also supported by structural conjunctions (Oishi et al., 2018, Wang et al., 2016). Consistent with these findings, our results supported the abnormal interactions between the two streams in patients with amblyopia and further revealed their associations with stereopsis defects.

Notably, our results emphasized the crucial roles of higher visual hierarchy (e.g. V3d, V3A, V3B, LO1, and LO2) in stereoscopic deficits in amblyopia. We can explain these findings in the following two ways. On the one hand, previous studies showed that deficits may increase as information goes up the hierarchy (Muckli et al., 2006, Mendola et al., 2018). On the other hand, the neural processing of stereopsis progressively improves in the higher-tier visual cortex, where the proportion of disparity-sensitive/insensitive neurons (Backus et al., 2001) and the degree of binocular correspondence (Chen et al., 2017, Verhoef et al., 2016) are higher.

### 4.3 Abnormal feedforward/feedback connections associated with stereopsis defects in patients with amblyopia

Compared with controls, the results of the cross-sectional dataset showed that the EC values of feedforward connections were mainly more negative, while the EC values of feedback connections were mainly more positive in patients with amblyopia. The more positive EC values could be interpreted as a more excitatory or less inhibitory influence. In contrast, the more negative ECs could be interpreted as a less excitatory or more inhibitory influence (Dijkstra et al., 2017). Notably, these findings could only suggest a net influence from one brain region to another, rather than suggesting the property of neurons (Bastos et al., 2012, Zeidman et al., 2019a). In general, feedforward connections are generally considered excitatory, while feedback connections are generally considered inhibitory (Bastos et al., 2012). Therefore, our results may suggest a weaker excitatory influence of feedforward connections and weaker inhibitory influence of feedback connections in the visual network of patients with amblyopia. The reduced excitatory influence of feedforward may be caused by abnormal visual experience in early life. Feedbacks deliver predictions, helping higher-level brain regions to interpret and reduce prediction errors in lower-level brain regions (Bastos et al., 2012). Therefore, the more negative inhibition of feedback connections may reflect the reduced predictive effect of higher-level cortical regions on lower cortical regions. Evidence from rodents provided support for our results, reporting that abnormal visual experience during the critical period could cause the imbalance of excitatory/inhibitory neurons, and unreliable and noisy excitatory drive may otherwise lead to a random strengthening and weakening of synaptic connections, which could impede the formation of the adequate circuitry necessary to process sensory stimuli (Levelt & Hübener, 2012). Two studies have reported abnormal feedforward and feedback in patients with amblyopia (Dai et al., 2021, Li et al., 2011). However. Li et al. (2011) found that both feedforward and feedback connections were equally weakened (Li et al., 2011), while Dai et al. (2021) found prominent abnormalities in feedback connections compared to feedforward connections (Dai et al., 2021). The differences in results may be attributable to the different ROIs selected. We included more detailed and complete ROIs in the visual network compared to Li et al. 2011, while Dai et al. 2021 included ROIs across the whole brain range. Furthermore, this study and Dai et al. (2021) compared patients with amblyopia and healthy controls, while Li et al. (2011) compared differences between the amblyopic eye and the fellow eye (non-amblyopic eye) in patients with amblyopia. However, the fellow eyes are not equal to the normal eyes even when they manifest normal visual acuities.

In addition, the results of the longitudinal dataset showed mainly more positive EC values in feedforward connections and decreased EC values in feedback connections in patients with amblyopia after perceptual learning. These changes may suggest increased excitatory influences in feedforward and increased inhibitory influences in feedback. Animal studies have concluded that mediating the balance of excitation and inhibition could be critical for recovery from amblyopia (Baroncelli et al., 2011), and the change in the excitatory/inhibitory balance in patients with amblyopia after perceptual learning therapy may be induced by GABAergic inhibition changes (Baroncelli et al., 2011). These findings provide a potential explanation for our results at the micro-level, while our results may provide support for these hypotheses at the macro level.

### 4.4 Limitations

This study has several limitations. First, the sample size of longitudinal data is small. Therefore, we adopted a strict threshold of Pp > 0.99 and focused on the most consistent findings with the cross-sectional study to increase the reliability of the results. Second, patients included in the two datasets were different in age, e.g. from 18 to 37 in the cross-sectional dataset and from 12 to 23 in the longitudinal dataset. However, the development of the visual system (Leat et al., 2001) and stereopsis (Romano et al., 1975) have been nearly fully developed by the age of 9, and aging will not affect stereopsis until age 60 (Garnham, 2006, Haegerstrom-Portnoy et al., 1999, Lee and Koo, 2005, Zaroff et al., 2003). Damage to the brain from amblyopia depends on abnormal visual experience in early life rather than worsening with age (Hensch, 2005, Pizzorusso et al., 2002). In addition, stereopsis deficiency could be effectively improved even in adult patients with amblyopia (Chopin et al., 2021, Ding and Levi, 2011, Liu and Zhang, 2019, Lunghi et al., 2019, Vedamurthy et al., 2015, Xi et al., 2014). Furthermore, in our study, we obtained similar results from PEB analyses with and without age as covariates, suggesting that the age differences in our two datasets may not influence the reliability of the results. Third, different head coils were used in the two datasets. This was because we collected the cross-sectional dataset about five years later than the longitudinal dataset and the head coil was updated to obtain a better signal-to-noise ratio and artifact control capability. Fourth, we did not conduct subgroup analysis in different subtypes of patients with amblyopia due to a limited subgroup sample size. It is interesting to research whether there are differences in the neural mechanisms of stereoscopic impairment among different subtypes of amblyopia. However, compared with the subtype differences, the present study preferred to focus on the neuroimaging differences due to the degree of stereoscopic defects. Finally, we obtained only resting-state fMRI data without external visual stimuli. Although there seems to be no significant difference in ECs of the human visual network between resting and task-related states (Zhao et al., 2020), the EC pattern in the resting state may represent the intrinsic states of the amblyopic brain and could be of reference significance to EC during stereoscopic stimulation. Future studies are required to verify our results using fMRI scanning with a stereoscopic task.

## 5 Conclusion

In summary, convergent results from our cross-sectional and longitudinal datasets proved that EC from V2v to LO2 was associated with stereopsis defects in patients with amblyopia. The abnormalities of the early visual cortex are not sufficient to explain the stereoscopic defects in patients with amblyopia. Instead, the higher-order dorsal (V3d, V3A, V3B) and ventral visual cortices (LO1, LO2) could serve as key nodes in the abnormal EC network contributing to the stereopsis defects of patients with amblyopia. Our research contributes to understanding the potential neural mechanism of stereopsis defects in patients with amblyopia and provides candidate targets for focused stimulus interventions to enhance the effectiveness of clinical treatment for the improvement of stereopsis deficiency.

## 6 Data Availability Statement

The data supporting the findings of this study are available on reasonable request from the corresponding author.

## Funding

This work was supported by the National Natural Science Foundation of China (82070996) and the Sichuan University postdoctoral interdisciplinary Innovation Fund (0040204153259).

## CRediT authorship contribution statement

**Xia Chen:** Conceptualization, Data curation, Formal analysis, Investigation, Methodology, Resources, Visualization, Writing – original draft. **Meng Liao:** Conceptualization, Investigation, Resources, Project administration, Supervision, Writing – review & editing. **Ping Jiang:** Conceptualization, Project administration, Software, Methodology, Writing – review & editing, Funding acquisition, Resources, Supervision. **Huaiqiang Sun:** Data curation, Resources. **Longqian Liu:** Conceptualization, Project administration, Funding acquisition, Resources, Supervision. **Qiyong Gong:** Conceptualization, Methodology, Software, Resources.

## Declaration of Competing Interest

The authors declare that they have no known competing financial interests or personal relationships that could have appeared to influence the work reported in this paper.
