# Supplementary material for: Abnormal effective connectivity in visual cortices underlies stereopsis defects in amblyopia
Source: Neuroimage Clin. 2022 Apr 8;34:103005. doi: 10.1016/j.nicl.2022.103005 (PMC9011166; doi:10.1016/j.nicl.2022.103005)
Supplement: Supplementary data 1 [file mmc1.docx]

Abnormal effective connectivity in visual cortices underlies stereopsis defects in amblyopia

Supplementary Materials

# Supplementary Methods

## Preprocessing using fMRIPrep of the cross-sectional dataset

Results included in this manuscript come from preprocessing performed using fMRIPrep 20.2.1 (Esteban, Markiewicz, et al. (2018); Esteban, Blair, et al. (2018); RRID: SCR_016216), which is based on Nipype 1.5.1 (Gorgolewski et al. (2011); Gorgolewski et al. (2018); RRID:SCR_002502).

### Anatomical data preprocessing

The T1-weighted (T1w) image was corrected for intensity non-uniformity (INU) with N4BiasFieldCorrection (Tustison et al. 2010), distributed with ANTs 2.3.3 (Avants et al. 2008, RRID:SCR_004757), and used as T1w-reference throughout the workflow. The T1w-reference was then skull-stripped with a Nipype implementation of the antsBrainExtraction.sh workflow (from ANTs), using OASIS30ANTs as target template. Brain tissue segmentation of cerebrospinal fluid (CSF), white-matter (WM) and gray-matter (GM) was performed on the brain-extracted T1w using fast (FSL 5.0.9, RRID: SCR_002823, Zhang, Brady, and Smith 2001). Brain surfaces were reconstructed using recon-all (FreeSurfer 6.0.1, RRID:SCR_001847, Dale, Fischl, and Sereno 1999), and the brain mask estimated previously was refined with a custom variation of the method to reconcile ANTs-derived and FreeSurfer-derived segmentations of the cortical gray-matter of Mindboggle (RRID:SCR_002438, Klein et al. 2017). Volume-based spatial normalization to two standard spaces (MNI152NLin6Asym, MNI152NLin2009cAsym) was performed through nonlinear registration with antsRegistration (ANTs 2.3.3), using brain-extracted versions of both T1w reference and the T1w template. The following templates were selected for spatial normalization: FSL’s MNI ICBM 152 non-linear 6th Generation Asymmetric Average Brain Stereotaxic Registration Model [Evans et al. (2012), RRID: SCR_002823; TemplateFlow ID: MNI152NLin6Asym], ICBM 152 Nonlinear Asymmetrical template version 2009c [Fonov et al. (2009), RRID: SCR_008796; TemplateFlow ID: MNI152NLin2009cAsym].

### Functional data preprocessing

For each of the 2 BOLD runs found per subject (across all tasks and sessions), the following preprocessing was performed. First, a reference volume and its skull-stripped version were generated by aligning and averaging 1 single-band references (SBRefs). A B0-nonuniformity map (or fieldmap) was estimated based on two (or more) echo-planar imaging (EPI) references with opposing phase-encoding directions, with 3dQwarp Cox and Hyde (1997) (AFNI 20160207). Based on the estimated susceptibility distortion, a corrected EPI (echo-planar imaging) reference was calculated for a more accurate co-registration with the anatomical reference. The BOLD reference was then co-registered to the T1w reference using bbregister (FreeSurfer) which implements boundary-based registration (Greve and Fischl 2009). Co-registration was configured with six degrees of freedom. Head-motion parameters with respect to the BOLD reference (transformation matrices, and six corresponding rotation and translation parameters) are estimated before any spatiotemporal filtering using mcflirt (FSL 5.0.9, Jenkinson et al. 2002). First, a reference volume and its skull-stripped version were generated using a custom methodology of fMRIPrep. The BOLD time-series (including slice-timing correction when applied) were resampled onto their original, native space by applying a single, composite transform to correct for head-motion and susceptibility distortions. These resampled BOLD time-series will be referred to as preprocessed BOLD in original space, or just preprocessed BOLD. The BOLD time-series were resampled into standard space, generating a preprocessed BOLD run in MNI152NLin6Asym space. First, a reference volume and its skull-stripped version were generated using a custom methodology of fMRIPrep. Several confounding time-series were calculated based on the preprocessed BOLD: framewise displacement (FD), DVARS and three region-wise global signals. FD was computed using two formulations following Power (absolute sum of relative motions, Power et al. (2014)) and Jenkinson (relative root mean square displacement between affines, Jenkinson et al. (2002)). FD and DVARS are calculated for each functional run, both using their implementations in Nipype (following the definitions by Power et al. 2014). The three global signals are extracted within the CSF, the WM, and the whole-brain masks. Additionally, a set of physiological regressors were extracted to allow for component-based noise correction (CompCor, Behzadi et al. 2007). Principal components are estimated after high-pass filtering the preprocessed BOLD time-series (using a discrete cosine filter with 128s cut-off) for the two CompCor variants: temporal (tCompCor) and anatomical (aCompCor). tCompCor components are then calculated from the top 2% variable voxels within the brain mask. For aCompCor, three probabilistic masks (CSF, WM and combined CSF+WM) are generated in anatomical space. The implementation differs from that of Behzadi et al. in that instead of eroding the masks by 2 pixels on BOLD space, the aCompCor masks are subtracted a mask of pixels that likely contain a volume fraction of GM. This mask is obtained by dilating a GM mask extracted from the FreeSurfer’s aseg segmentation, and it ensures components are not extracted from voxels containing a minimal fraction of GM. Finally, these masks are resampled into BOLD space and binarized by thresholding at 0.99 (as in the original implementation). Components are also calculated separately within the WM and CSF masks. For each CompCor decomposition, the k components with the largest singular values are retained, such that the retained components’ time series are sufficient to explain 50 percent of variance across the nuisance mask (CSF, WM, combined, or temporal). The remaining components are dropped from consideration. The head-motion estimates calculated in the correction step were also placed within the corresponding confounds file. The confound time series derived from head motion estimates and global signals were expanded with the inclusion of temporal derivatives and quadratic terms for each (Satterthwaite et al. 2013). Frames that exceeded a threshold of 0.5 mm FD or 1.5 standardised DVARS were annotated as motion outliers. All resamplings can be performed with a single interpolation step by composing all the pertinent transformations (i.e. head-motion transform matrices, susceptibility distortion correction when available, and co-registrations to anatomical and output spaces). Gridded (volumetric) resamplings were performed using antsApplyTransforms (ANTs), configured with Lanczos interpolation to minimize the smoothing effects of other kernels (Lanczos 1964). Non-gridded (surface) resamplings were performed using mri_vol2surf (FreeSurfer).

Finally, two preprocessed BOLD runs with two opposite phase encoding directions were concatenated and smoothed with a 6 mm full width at half maximum (FWHM) Gaussian kernel.

## ROI definition

**Table S1**

**ROI definition**

| Dorsal | | | Ventral | | |
| --- | --- | --- | --- | --- | --- |
| ROI | Coordinates in MNI | | ROI | Coordinates in MNI | |
|  | L: x, y, z | R: x, y, z |  | L: x, y, z | R: x, y, z |
| V1d | -8, -89, 4 | 11, -87, 7 | V1v | -6, -82, -3 | 9, -80, -1 |
| V2d | -10, -91, 12 | 14, -88, 15 | V2v | -10, -78, -8 | 10, -76, -7 |
| V3d | -17, -89, 15 | 21, -86, 17 | V3v | -18, -76, -10 | 18, -72, -8 |
| V3A | -18, -85, 23 | 22, -82, 27 | hV4 | -27, -77, -12 | 29, -76, -11 |
| V3B | -29, -84, 15 | 34, -80, 17 | LO1 | -33, -83, 8 | 36, -81, 9 |
| hMT | -45, -74, 7 | 48, -67, 8 | LO2 | -40, -80, 7 | 42, -75, 8 |
| MST | -47, -66, 8 | 47, -60, 7 |  |  |  |
| IPS0 | -25, -75, 31 | 29, -73, 32 |  |  |  |

**Abbreviations:** ROI: region of interest; MNI: Montreal Neurological Institute; L: left hemisphere; R: right hemisphere; V1: primary visual cortex; V2: secondary visual cortex; V3: visual area V3; (v: ventral; d: dorsal); hV4: human visual region V4; hMT: human middle temporal region; MST: medial superior temporal area; IPS: intraparietal sulcus; LO: lateral occipital.

## Supplementary information about DCM and PEB

The spectral DCM contained endogenous connectivity and was quantified by ‘A matrix’ parameters because no experimental conditions were specified in the model. First, the BOLD signal was extracted for 14 predefined ROIs using a general linear model with six head motion parameters added to the general linear model as nuisance regressors. Second, we specified a fully connected DCM model (each node connects to itself and all other nodes) consisting of 14 ROIs. Then, subject-level model estimation based on standard variational Bayes procedures (variational Laplace) under the frequency domain was performed. The convolution kernel of the model was converted into a spectrum and expressed in the frequency domain. This approximate Bayesian inference method can quickly estimate the connectivity parameters and logarithmic model evidence of first-level DCMs and effectively optimize the posterior probability of model parameters.

The PEB framework is superior to classical statistics in that it includes the expected values and covariates of parameters at the group level and avoids the problem of multiple comparisons. In the Bayesian framework used in PEB analysis, there is no indicator similar to the typical statistical significance level. Instead, Bayesian posterior probability (Bayesian-Pp) is used as an indicator of confidence. The higher Bayesian-Pp indicates greater confidence.

# Supplementary Results

## Results of PEB 1: group differences between patients with amblyopia and healthy controls in the cross-sectional dataset


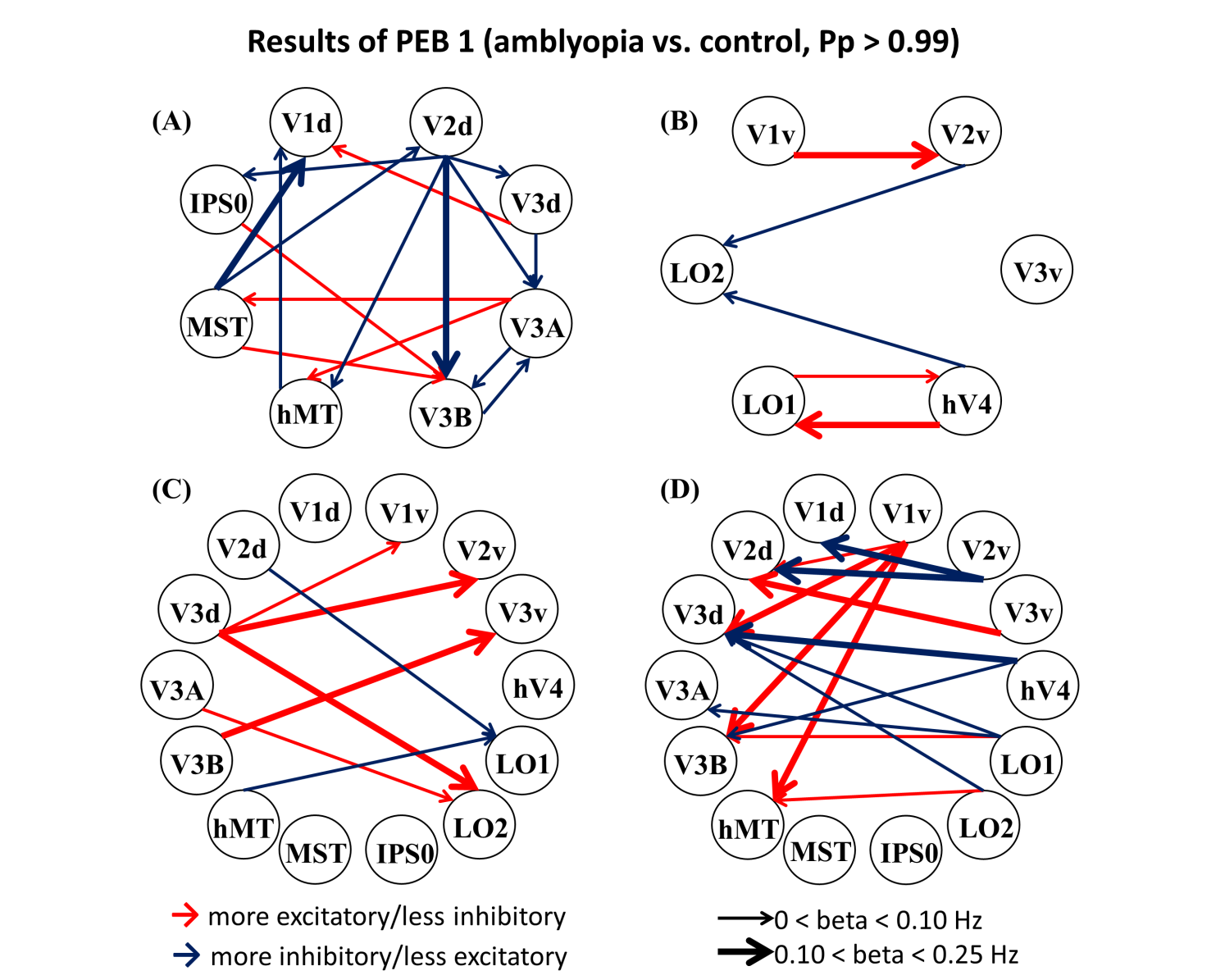


**Fig. S1.** **Results of PEB 1 (with education years, age, and sex as covariates): group differences in the cross-sectional dataset (amblyopia vs. control, Pp > 0.99).** This figure presents abnormal connections in patients with amblyopia compared with healthy controls. Only results with very strong evidence (Pp > 0.99) are depicted. Lines with arrows represent connections **(A)** within the dorsal visual stream; **(B)** within the ventral visual stream; **(C)** From the dorsal to ventral visual stream; and **(D)** from the ventral to dorsal visual stream. The arrows indicate the direction of the connection. Red lines denote increased connections in patients with amblyopia; while blue lines denote decreased connections in patients with amblyopia. Lines are scaled by the effect size of PEB 1 from 0 to 0.25 Hz. **Abbreviations:** PEB: parametric empirical Bayes; Pp: posterior probability.


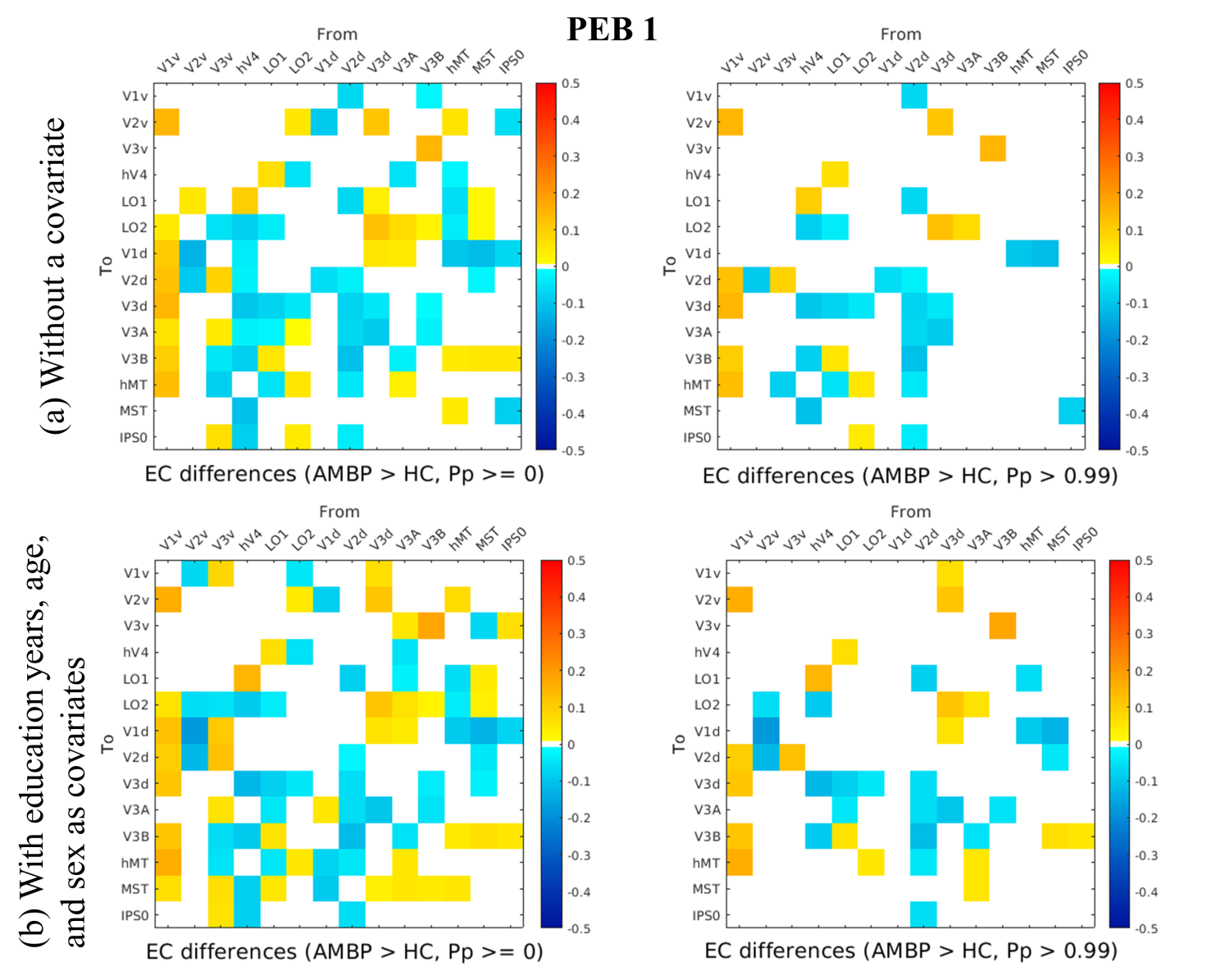


**Fig. S2. Results of PEB 1 with and without covariates: (a)** The upper panel shows the results of PEB 1 without a covariate. **(b)** The bottom panel presents the results of PEB 1 with education years, age, and sex as covariates of no interest. The left side shows all “nontrivial” results (Pp >= 0). The right side depicts results with “very strong evidence” (Pp > 0.99). The source regions are shown as row headers, while the target regions are shown as column headers. The warm color represents an excitatory influence from the source region to the target region, while the cold color represents an inhibitory influence from the source region to the target region. **Abbreviations:** PEB: parametric empirical Bayes; EC: effective connectivity; Pp: posterior probability; AMBP: amblyopia; HC: healthy control.

## Results of PEB 2: the relationship between EC and TNO values in the cross-sectional dataset

**
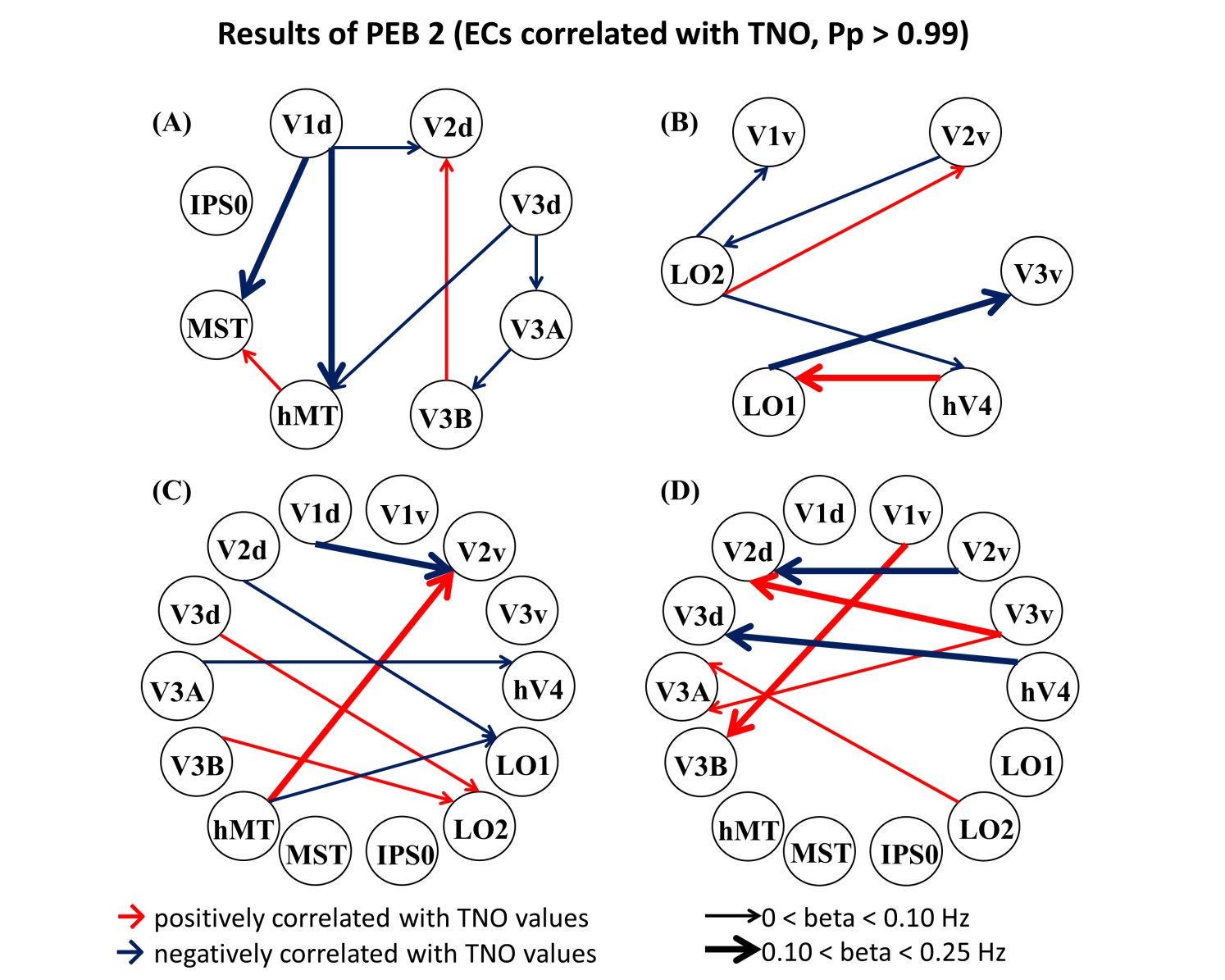
**

**Fig. S3. Results of PEB 2: the relationship between ECs and TNO values in the cross-sectional dataset (with the group, education years, age, and sex as covariates, Pp > 0.99).** This picture shows ECs related to TNO values. Only results with very strong evidence (Pp > 0.99) are depicted. Lines with arrows represent ECs **(A)** within the dorsal visual stream; **(B)** within the ventral visual stream; **(C)** from the dorsal to ventral visual stream; and **(D)** from the ventral to dorsal visual stream. The arrows indicate the direction of the connection. Red lines denote ECs positively related to TNO values; while blue lines denote ECs negatively related to TNO values. Lines are scaled by the effect size of PEB 2 from 0 to 0.25 Hz. **Abbreviations:** PEB: parametric empirical Bayes; EC: effective connectivity; Pp: posterior probability; TNO: the Netherlands Organisation for applied scientific research, refers to TNO stereo test here.


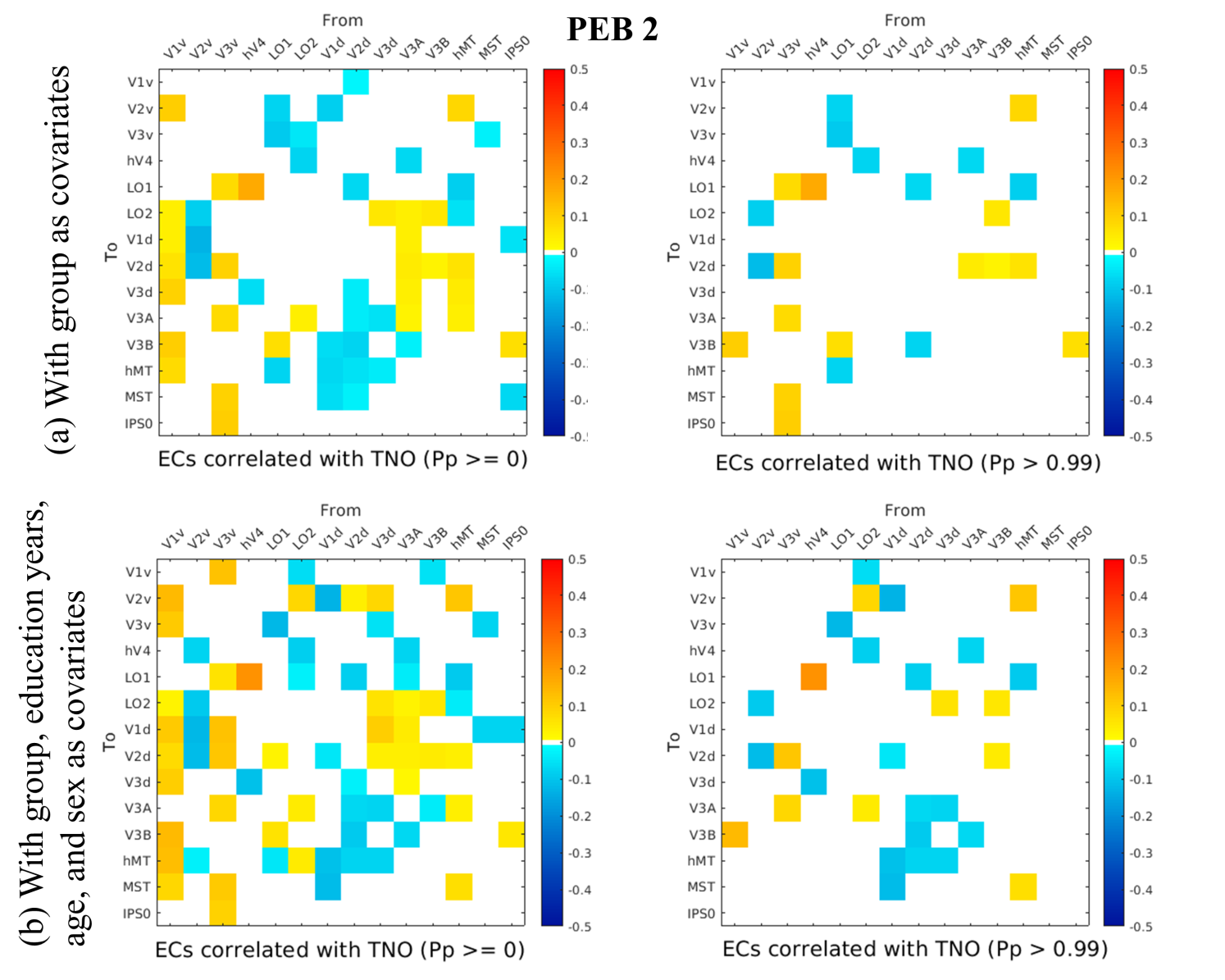


**Fig. S4. Results of PEB 2 with and without covariates: (a)** The upper panel shows the results of PEB 2 with the group as covariates of no interest. **(b)** The bottom panel presents the results of PEB 2 with the group, education years, age, and sex as covariates of no interest. The left side shows all “nontrivial” results (Pp >= 0). The right side depicts results with “very strong evidence” (Pp > 0.99). The source regions are shown as row headers, while the target regions are shown as column headers. The warm color represents an excitatory influence from the source region to the target region, while the cold color represents an inhibitory influence from the source region to the target region. **Abbreviations:** PEB: parametric empirical Bayes; EC: effective connectivity; Pp: posterior probability; TNO: the Netherlands Organisation for applied scientific research, refers to TNO stereo test here.

## Results of PEB 3: group differences between post- and pre-treatment patients with amblyopia in the longitudinal dataset

**
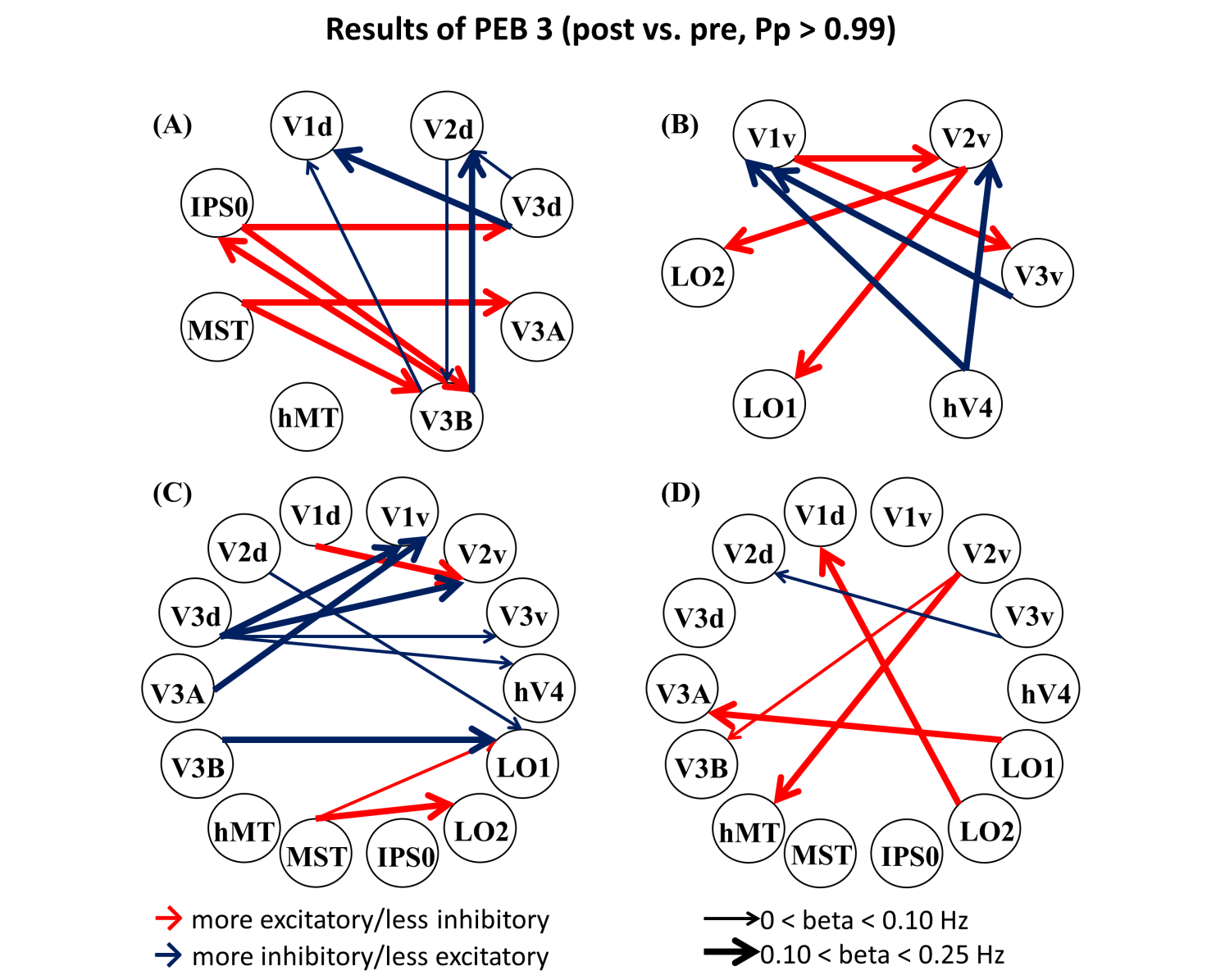
**

**Fig. S5. Results of PEB 3 (with education years, age, and sex as covariates): group differences in the longitudinal dataset (post-treatment vs. pre-treatment, Pp > 0.99).** Lines with arrows represent connections **(A)** within the dorsal visual stream; **(B)** within the ventral visual stream; **(C)** from the dorsal to ventral visual stream; and **(D)** from the ventral to dorsal visual stream. The arrows indicate the direction of the connection. Red lines denote increased connectivity in the post-treatment group; while blue lines denote decreased connectivity in the post-treatment group. Lines are scaled by the effect size of PEB 3 from 0 to 0.25 Hz. **Abbreviations:** PEB: parametric empirical Bayes; Pp: posterior probability.

**
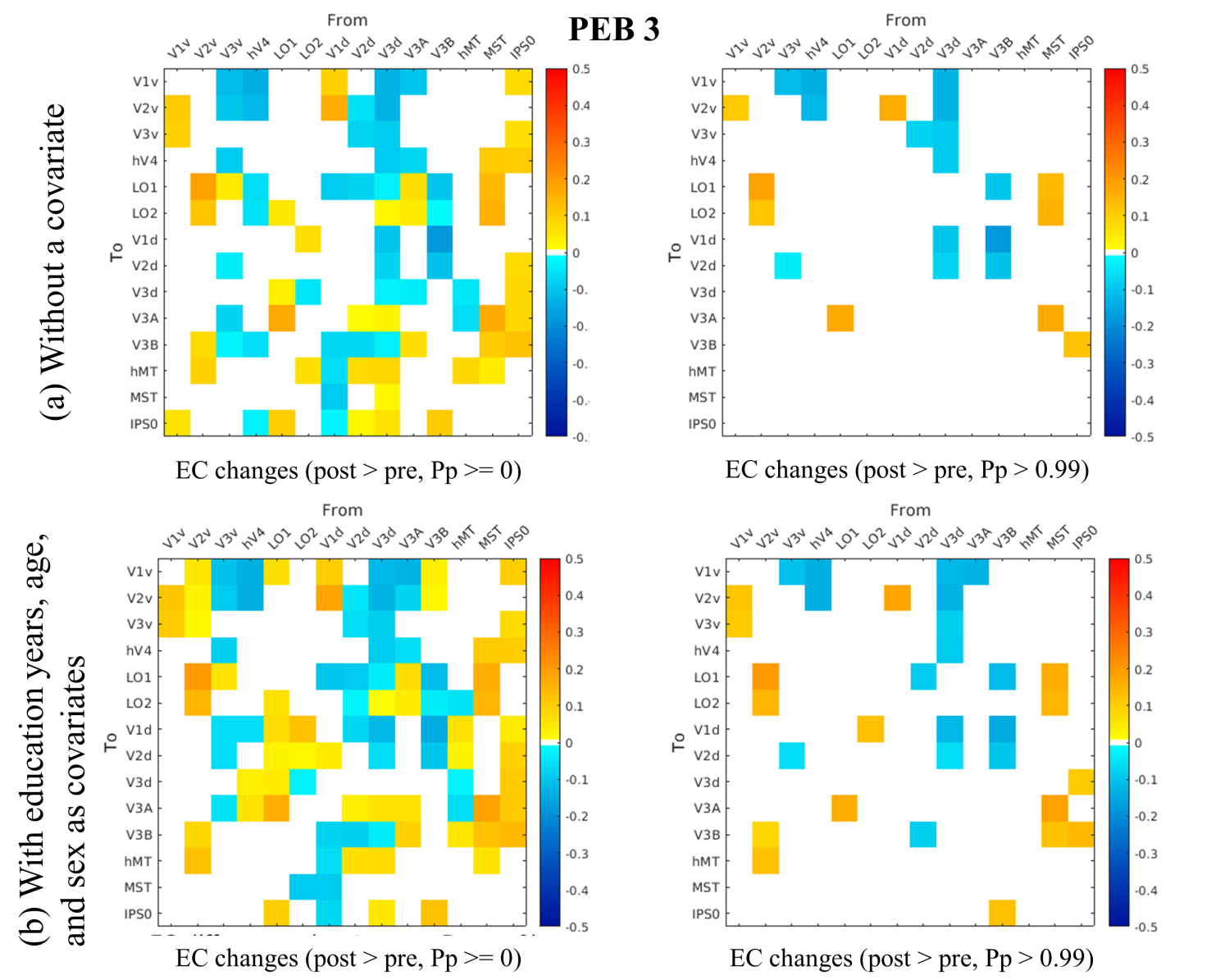
**

**Fig. S6. Results of PEB 3 with and without covariates: (a)** The upper panel shows the results of PEB 3 without a covariate. **(b)** The bottom panel presents the results of PEB 3 with education years, age, and sex as covariates of no interest. The left side shows all “nontrivial” results (Pp >= 0). The right side depicts results with “very strong evidence” (Pp > 0.99). The source regions are shown as row headers, while the target regions are shown as column headers. The warm color represents an excitatory influence from the source region to the target region, while the cold color represents an inhibitory influence from the source region to the target region. **Abbreviations:** PEB: parametric empirical Bayes; EC: effective connectivity; Pp: posterior probability.

## Results of PEB 4: the relationship between EC and TNO values in the longitudinal dataset

**
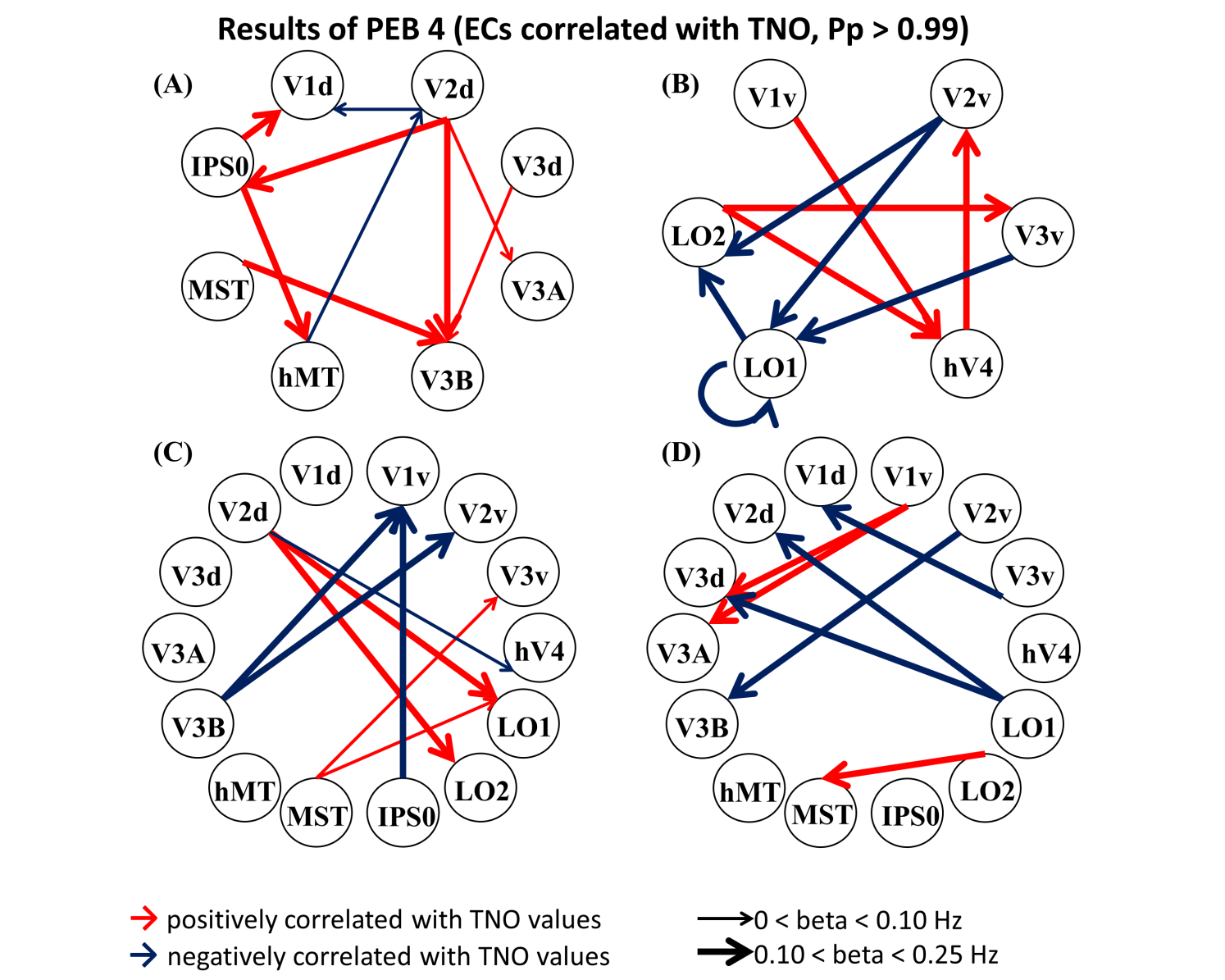
**

**Fig. S7. Results of PEB 4: the relationship between ECs and TNO values in the longitudinal dataset (with the group, education years, age, and sex as covariates, Pp > 0.99).** This figure showed ECs correlated with TNO values. Lines with arrows represent ECs **(A)** within the dorsal visual stream; **(B)** within the ventral visual stream; **(C)** from the dorsal to ventral visual stream; and **(D)** from the ventral to dorsal visual stream. The arrows indicate the direction of the connection. Red lines denote ECs positively correlated with TNO values; while blue lines denote ECs negatively correlated with TNO values. Lines are scaled by the effect size of PEB 4 from 0 to 0.25 Hz. **Abbreviations:** PEB: parametric empirical Bayes; EC: effective connectivity; Pp: posterior probability; TNO: the Netherlands Organisation for applied scientific research, refers to TNO stereo test here.


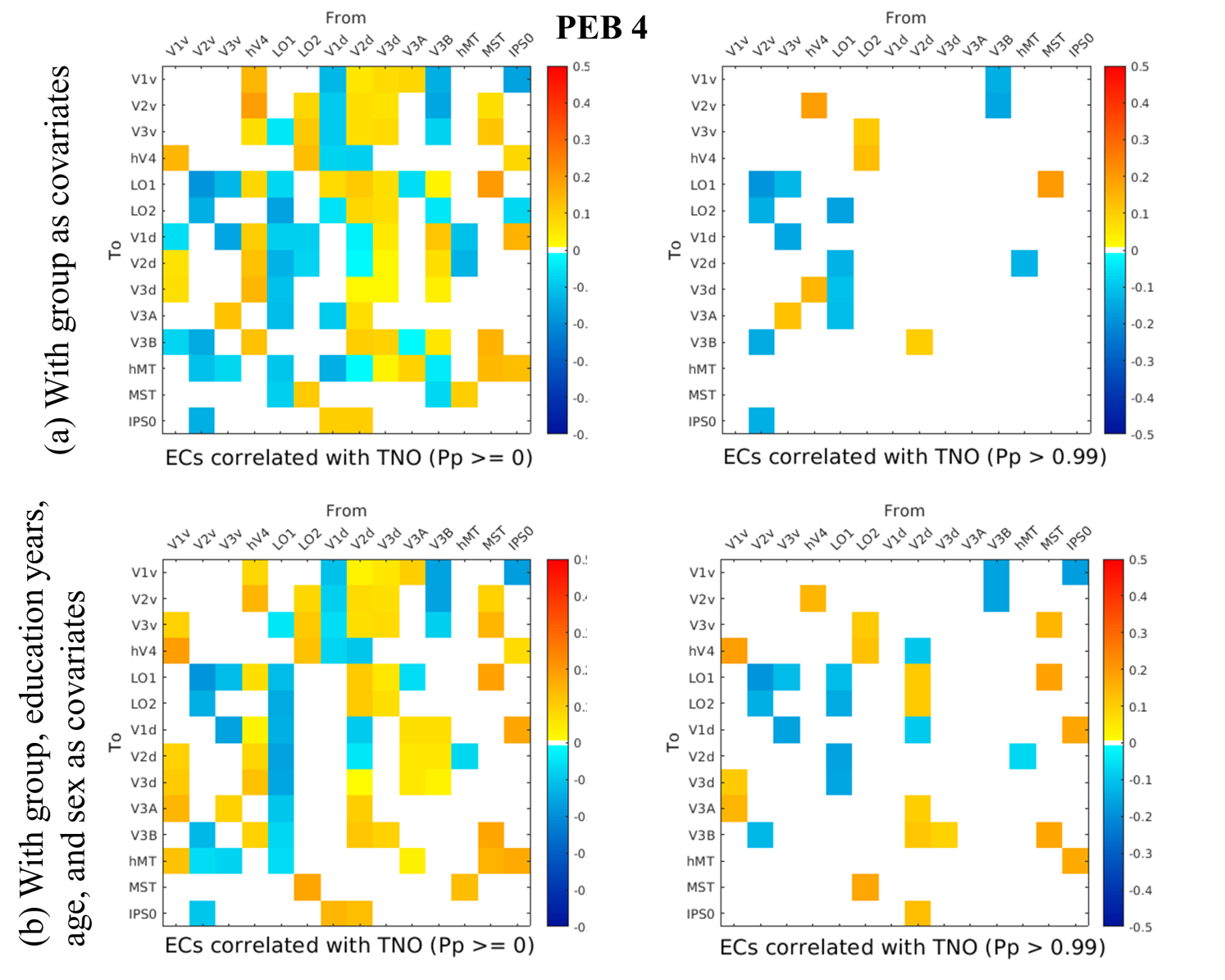


**Fig. S8. Results of PEB 4 with and without covariates: (a)** The upper panel shows the results of PEB 4 with the group as covariates of no interest. **(b)** The bottom panel presents the results of PEB 4 with the group, education years, age, and sex as covariates of no interest. The left side shows all “nontrivial” results (Pp >= 0). The right side depicts results with “very strong evidence” (Pp > 0.99). The source regions are shown as row headers, while the target regions are shown as column headers. The warm color represents an excitatory influence from the source region to the target region, while the cold color represents an inhibitory influence from the source region to the target region. **Abbreviations:** PEB: parametric empirical Bayes; EC: effective connectivity; Pp: posterior probability; TNO: the Netherlands Organisation for applied scientific research, refers to TNO stereo test here.

### References

Abraham, Alexandre, Fabian Pedregosa, Michael Eickenberg, Philippe Gervais, Andreas Mueller, Jean Kossaifi, Alexandre Gramfort, Bertrand Thirion, and Gael Varoquaux. 2014. “Machine Learning for Neuroimaging with Scikit-Learn.” Frontiers in Neuroinformatics 8. <https://doi.org/10.3389/fninf.2014.00014>.

Avants, B.B., C.L. Epstein, M. Grossman, and J.C. Gee. 2008. “Symmetric Diffeomorphic Image Registration with Cross-Correlation: Evaluating Automated Labeling of Elderly and Neurodegenerative Brain.” Medical Image Analysis 12 (1): 26–41. <https://doi.org/10.1016/j.media.2007.06.004>.

Behzadi, Yashar, Khaled Restom, Joy Liau, and Thomas T. Liu. 2007. “A Component Based Noise Correction Method (CompCor) for BOLD and Perfusion Based fMRI.” NeuroImage 37 (1): 90–101. <https://doi.org/10.1016/j.neuroimage.2007.04.042>.

Cox, Robert W., and James S. Hyde. 1997. “Software Tools for Analysis and Visualization of fMRI Data.” NMR in Biomedicine 10 (4-5): 171–78. [https://doi.org/10.1002/(SICI)1099-1492(199706/08)10:4/5<171::AID-NBM453>3.0.CO;2-L](https://doi.org/10.1002/(SICI)1099-1492(199706/08)10:4/5%3c171::AID-NBM453%3e3.0.CO;2-L).

Dale, Anders M., Bruce Fischl, and Martin I. Sereno. 1999. “Cortical Surface-Based Analysis: I. Segmentation and Surface Reconstruction.” NeuroImage 9 (2): 179–94. <https://doi.org/10.1006/nimg.1998.0395>.

Esteban, Oscar, Ross Blair, Christopher J. Markiewicz, Shoshana L. Berleant, Craig Moodie, Feilong Ma, Ayse Ilkay Isik, et al. 2018. “FMRIPrep.” Software. Zenodo. <https://doi.org/10.5281/zenodo.852659>.

Esteban, Oscar, Christopher Markiewicz, Ross W Blair, Craig Moodie, Ayse Ilkay Isik, Asier Erramuzpe Aliaga, James Kent, et al. 2018. “fMRIPrep: A Robust Preprocessing Pipeline for Functional MRI.” Nature Methods. <https://doi.org/10.1038/s41592-018-0235-4>.

Evans, AC, AL Janke, DL Collins, and S Baillet. 2012. “Brain Templates and Atlases.” NeuroImage 62 (2): 911–22. <https://doi.org/10.1016/j.neuroimage.2012.01.024>.

Fonov, VS, AC Evans, RC McKinstry, CR Almli, and DL Collins. 2009. “Unbiased Nonlinear Average Age-Appropriate Brain Templates from Birth to Adulthood.” NeuroImage 47, Supplement 1: S102. <https://doi.org/10.1016/S1053-8119(09)70884-5>.

Gorgolewski, K., C. D. Burns, C. Madison, D. Clark, Y. O. Halchenko, M. L. Waskom, and S. Ghosh. 2011. “Nipype: A Flexible, Lightweight and Extensible Neuroimaging Data Processing Framework in Python.” Frontiers in Neuroinformatics 5: 13. <https://doi.org/10.3389/fninf.2011.00013>.

Gorgolewski, Krzysztof J., Oscar Esteban, Christopher J. Markiewicz, Erik Ziegler, David Gage Ellis, Michael Philipp Notter, Dorota Jarecka, et al. 2018. “Nipype.” Software. Zenodo. <https://doi.org/10.5281/zenodo.596855>.

Greve, Douglas N, and Bruce Fischl. 2009. “Accurate and Robust Brain Image Alignment Using Boundary-Based Registration.” NeuroImage 48 (1): 63–72. <https://doi.org/10.1016/j.neuroimage.2009.06.060>.

Jenkinson, Mark, Peter Bannister, Michael Brady, and Stephen Smith. 2002. “Improved Optimization for the Robust and Accurate Linear Registration and Motion Correction of Brain Images.” NeuroImage 17 (2): 825–41. <https://doi.org/10.1006/nimg.2002.1132>.

Klein, Arno, Satrajit S. Ghosh, Forrest S. Bao, Joachim Giard, Yrjö Häme, Eliezer Stavsky, Noah Lee, et al. 2017. “Mindboggling Morphometry of Human Brains.” PLOS Computational Biology 13 (2): e1005350. <https://doi.org/10.1371/journal.pcbi.1005350>.

Lanczos, C. 1964. “Evaluation of Noisy Data.” Journal of the Society for Industrial and Applied Mathematics Series B Numerical Analysis 1 (1): 76–85. <https://doi.org/10.1137/0701007>.

Power, Jonathan D., Anish Mitra, Timothy O. Laumann, Abraham Z. Snyder, Bradley L. Schlaggar, and Steven E. Petersen. 2014. “Methods to Detect, Characterize, and Remove Motion Artifact in Resting State fMRI.” NeuroImage 84 (Supplement C): 320–41. <https://doi.org/10.1016/j.neuroimage.2013.08.048>.

Satterthwaite, Theodore D., Mark A. Elliott, Raphael T. Gerraty, Kosha Ruparel, James Loughead, Monica E. Calkins, Simon B. Eickhoff, et al. 2013. “An improved framework for confound regression and filtering for control of motion artifact in the preprocessing of resting-state functional connectivity data.” NeuroImage 64 (1): 240–56. <https://doi.org/10.1016/j.neuroimage.2012.08.052>.

Tustison, N. J., B. B. Avants, P. A. Cook, Y. Zheng, A. Egan, P. A. Yushkevich, and J. C. Gee. 2010. “N4ITK: Improved N3 Bias Correction.” IEEE Transactions on Medical Imaging 29 (6): 1310–20. <https://doi.org/10.1109/TMI.2010.2046908>.

Zhang, Y., M. Brady, and S. Smith. 2001. “Segmentation of Brain MR Images Through a Hidden Markov Random Field Model and the Expectation-Maximization Algorithm.” IEEE Transactions on Medical Imaging 20 (1): 45–57. <https://doi.org/10.1109/42.906424>.
